# Improving the Safety–Performance Nexus: A Study on the Moderating and Mediating Influence of Work Motivation in the Causal Link between Occupational Health and Safety Management (OHSM) Practices and Work Performance in the Oil and Gas Sector

**DOI:** 10.3390/ijerph18105064

**Published:** 2021-05-11

**Authors:** Edmund Nana Kwame Nkrumah, Suxia Liu, David Doe Fiergbor, Linda Serwah Akoto

**Affiliations:** 1 School of Environment and Safety Engineering, Jiangsu University, 301 Xuefu Road, Jingkou District, Zhenjiang 212013, China; nkrunak@gmail.com; 2 School of Business, Pentecost University College, Accra P.O. Box KN 1739, Ghana; daviddoef@gmail.com; 3 School of Management, Jiangsu University, 301 Xuefu Road, Jingkou District, Zhenjiang 212013, China; Amalinda9@gmail.com

**Keywords:** occupational health, safety management, work motivation, work performance, task performance, oil and gas

## Abstract

The preventive systems required to ensure workers are protected from occupational accidents and injuries dwell heavily on effective occupational health and safety management (OHSM) systems and practices. In this study, the concepts of the job demand-resource model (JD-R), self-determination theory (SDT), and perceived organizational support for safety (POSS) theory were adopted to develop a holistic conceptual model that seeks to unravel moderating and mediating effects of work motivation on the causal link between OHSM practices and work performance in the oil and gas sector. The study measured OHSM practices from six distinct safety dimensional perspectives and work performance using a two-dimensional distinct construct that assesses different aspects of positive work behaviours. A quantitative research approach through the structural equation modelling analysis technique was applied. A total of 1310 participants were selected across three major organizations that represent downstream, upstream, and middle stream of the Ghanaian oil and gas sector. Respondents were recruited through stratified, purposive, and convenient sampling techniques. The findings from the path estimate through the SEM analysis suggested that OHSM practices positively and significantly influenced both safety performance and task performance of employees. However, OHSM practices indicated a higher positive significant influence on task performance than safety performance. The significant influence of OHSM practices on both task and safety performance was significantly moderated and partially mediated by work motivation, while both task performance and safety performance were significantly determined by work motivation. In this study, the dimensions for assessing work performance extend the performance theories established in previous literature, whereas the integrated multifaceted OHSM practices employed diverge from the traditional individualistic approach by providing insights into more flexible managerial practices that are employee-centred and outcome-oriented. The findings from this study address the need for organizations to appreciate the importance of managing workers’ perception of OHSM practices as a motivational drive that induces work performance.

## 1. Introduction

In the quest to unravel the mystery behind certain safety-related behaviours exhibited by workers, the factor that gained the most prominent research attention is effective health and safety management practices [1,2,3,4,5,6]. The concept has become quite prominent in contemporary management and has been hugely adopted by industries to manage and improve the quality of life among employees. As posited by Kahya [7] an unsafe working environment (i.e., including the use of old and weak machinery, chemical exposures, unnecessary sounds at the workplace, etc.) can cause serious health issues and affect the well-being of workers. These negative influences of a poor working environment on employees’ well-being can as well go a long way to affect their productivity and organization performance as a whole. Human behaviour is quite complex and dynamic [8], hence, their ability to understand, comply and perform in accordance with safety procedures and standards remains significant to success across all highly risky industries [9,10]. Improving workers’ performance in a highly risky industry, therefore, means that organizations must be very considerate in the effective administration of occupational health and safety management practices as it may predict positive working behaviour and organizational efficiency [11].

The production and operational activities of the oil and gas sector in Ghana, including both the onshore and offshore operations and as well as all phases of the operation chain comes with numerous catastrophic and hazardous elements (i.e., explosions, blowouts, fire outbreaks, hydrocarbon leakages, falling objects, and hydrogen sulphide emissions, etc.) that are likely to cause workplace accidents, occupational injuries and damage to properties or lives. It is therefore not surprising why safety within the oil and gas industry remains highly regulated, and heavily governed by rules and regulations. Thus, a high level of safety presupposes a high level of safety compliance and participation [12]. This position might therefore account for the reason why most studies have focused mostly on safety-related outcomes such as safety compliance and safety participation rather than work performance [13,14]. The basic analogy is that employees who comply and participate in safety-related activities are likely to observe safety rules and procedures whiles performing their work responsibilities [13]. However, studies have shown that these two recognized components of safety performance, are significant leading indicators of injuries and incidents in the process industries, [15], and not necessarily the work performance of employees. In other studies, the safety performance variable was viewed in the context of work performance by extending the scope to include the contextual performance of workers [16].

The large workforce of an organization in the oil and gas industry remains a major factor that drives organizational performance, hence, the importance of sustaining their quality of life at the workplace is a necessity that is likely to improve their work performance. This current study, therefore, did not view safety performance only as one of the most important variables in high-risk industries but as well considers safety performance as a factor that is significantly affiliated to task performance. Thus, the work performance theory [16], which extended safety performance to include contextual performance, was expanded to include task performance and termed as the work performance construct. This is in line with the analogy purported by previous studies that assumed safety performance as a subset of organizational performance and productivity [17,18]. In this case, the study is holistically focusing on measuring the causal relationship between OHSM practices and a work performance construct that integrates both safety performance and task performance. More so, ascertaining the mediating and moderating influence of work motivation on the causal relationship between OHSM practices and work performance remains an important phenomenon which this study seeks to understand.

Thus, the fundamental principle of improving work performance through occupational health and safety travels beyond OHSM practices, hence, the hypothesis for this study was built on a conceptual model that proposes work motivation as a conduit that is likely to improve the influence OHSM practices may likely have on work performance among employees. This causal relationship between OHSM practices and performance per the accounts of literature can be explained by several factors, yet no studies have considered studying the mediation and moderation effect of work motivation on this interesting relationship. The intriguing question that needs to be examined is, do people merely perform their tasks and practice safe behaviour only because they are extrinsically or intrinsically motivated?

### 1.1. Definition of Terms

#### 1.1.1. Occupational Health and Safety Management (OHSM) Practices

Globally, organizations have responsibilities towards the protection of human life hence the management of risk and hazards at the workplace is an essential element of operational activities. Irrespective of this preposition, risk and hazards management among organizations, precisely highly risky industries, differ in administration and efficiency, hence, achieving functioning and systematic OHSM practices as well differs [19,20]. Without contentions, it can be argued that systematic and functioning OHSM systems and practices among organizations save lives and improve the quality of life of workers [21]. However, what determines an efficient OHSM practice even in accordance to literature differs in context and dimensions [20,22,23] due to several challenges such as the lack of commitment [21], lack of knowledge [24], lower financial resources [24,25], and giving priorities to production and operational activities whiles completely ignoring the safety of workers [19,20].

Be as it may be, one of the most recognized and significant OHSM practice highlighted by previous studies is the degree of management support and commitment for successful safety performance [13,26,27,28,29]. Thus, organizations with a strong management commitment towards improving OHSM practices are more likely to decline OHSM related accidents and injuries while at the same time experience positive job outcomes [30,31]. The OHSM practices employed in this study, therefore, focuses more on safety guidelines, principles, and practices that are built on management safety commitment and leadership, with much focus on safety practices that will improve the health, wellbeing, and behaviours of employees. These include workers’ perception of work safety, management commitment to safety, employee satisfaction with safety programs and policies, the availability and use of plant and Personal Protection Equipment (PPEs), dealing with organizational hazards, and health and safety training programs initiated by the organization. Thus, the study strives to achieve a collective and holistic approach to OHSM practices that diverges from the traditional individualistic approach by providing insights into more flexible managerial practices that are employee-centred and outcome-oriented.

Work safety in this study focuses on the relationship between the worker, the task, the tools, and the work environment by measuring the degree to which workers are informed on work-related hazards and risk exposures affiliated with the job.

As demonstrated by Perceived Organizations Support for Safety (POSS) theory, management safety commitment as a variable in this study refers to Management exhibition of commitment towards safety management practices by investing interest in improving employees’ safety-related issues at work.

Safety programs and policies in this study measures workers’ perceptions concerning their satisfaction with the organization’s safety programs and policies whiles organizational hazards focuses on the willingness and ability of the organization to learn, understand, and adapt its operations proactively by effectively examining incidents and near misses before an accident occurs.

Not least, Plant and Equipment/Personal Protection Equipment (PPEs) measures employees’ perception of the use of the right plants and equipment at the workplace. It assesses workers’ knowledge of organization plant and equipment and familiarity with the safety protocols associated with its usage. On the other hand, safety training focuses on measuring respondents’ perceptions of the degree to which they have received sufficient training regarding health and safety issues in their work environment.

#### 1.1.2. Work Performance

Abramis [32] defined work performance as any form of behaviour that contributes to the effective and efficient completion of work responsibilities that directly or indirectly contribute to organizational goals. The context of this definition highlights the significance of human resources as one of the organizational inputs that trigger performance, hence, their behaviour and attitudes towards work directly contribute significantly to organizational success. In the context of this study, the work performance theory was extended to integrate safety and task Performance among employees. In as much previous literature has shed light on the causal relationship between OHSM and safety performance, as well as workers’ behaviours, engagements, or wellbeing, little work has been done on scientifically questioning the inputs of task performance as an outcome of safety performance [16].

Task performance literally refers to the degree of effectiveness of work performance that feeds into the organizational goals and objectives [16]. Campbell, [33] explained task performance as the ability of workers to substantively perform their core mandates as expected by the organization. These include worker’s proficiency in job skills, job knowledge, work quantity, and work quality. Thus, employees’ task performance describes the performance of job responsibilities devoid of mistakes, handling of job demands, and taking the right decisions at all times [32].

On the other hand, safety performance refers to changes in work behaviours that are expected to prevent or reduce workplace accidents, occupational injuries, and illness. In most cases, some organizations adopt injury or accident logs to determine the safety performance of workers. This is mostly determined by the level of safety compliance and safety participation as propounded by [14]. Whiles Safety compliance refers to the level of employees’ adherence to safety rules, procedures, regulations, and standards related to their work, safety participation includes the level of employees’ involvement in safety programs, safety meetings, and helping others at work to adhere to safety standards [13]. Both dimensions of measuring safety performance focus on employee behaviours that are geared towards accident and injury prevention at the workplace.

#### 1.1.3. Work Motivation

The concept of work motivation has been widely discussed and explained in previous studies. Butkus and Green [34] carved the concept of motivation from the word “motivate” and defined work motivation as a means to persuade, push, and move to act in accordance to needs satisfaction. Be as the concept of motivation may be applied both in practice and theory, the concept basically entails understanding the factors that can trigger and induce an individual’s interest in his job [35]. Thus, motivation includes any work-related factors that can influence the maintenance of positive work behaviours expected to enhance productivity. This also means that managers have a role to play in providing the needed motivation factors that can psychologically trigger employee interest and directions towards goal achievement. This makes the concept of Work motivation a more complex and sensitive subject as it remains quite intriguing to know the specific factor that exactly motivates an employee to perform at work [36]. Hafiza et al. [37] however indicated that the work environment, working relationship, and job security are very good measurements of work motivation.

Previous studies relating to occupational health and safety management have focused on safety motivation, which remains a subset of motivation factors in itself [13,33,38,39], to explain the influence of OHSM on safety performance. The locus of this study diverges from these works, as it focuses on understanding the motivational factors, either extrinsic or intrinsic, that induce and energize employees to be involved in safety practices and as well task-related activities. Thus, the interest is to ascertain the factors that motivate the worker to willingly practice safety and task-related behaviour at work and ascertain how OHSM practices improve this level of motivation [13].

### 1.2. Hypothesis and Conceptual Framework

#### 1.2.1. The Causal Link between OHSM Practices and Work Performance

Previous studies have highly established that occupational health and safety issues are increasingly associated with both the operational efficiency, competitiveness of organizations and safety performance [28,40,41]. Recent studies, including Gopang et al. [42] showed that investment in OHSM moderately influences performances among Small and Medium Enterprises. A safe work environment positively predicts the psychological well-being of employees [43]. Ramazan et al. [44] revealed that the application of safety procedures, strategic risk mitigation techniques, application of safety rules, and management safety support significantly influences productivity levels among employees. Bottani et al. [45] found the adoption of safety management as a predictor of higher job performance among employees. They argued that the quality of efforts and inputs that employees invest in their jobs due to work safety is a major determinant of organization yield on operational outcomes. Other studies have as well attempted to link safety practices to numerous related safety outcomes, including occupational injuries and accident reduction, safety commitment, safety satisfaction, employee productivity, and other related safety events [14,31,46,47]. Occupational safety systems can, therefore, be considered by organizations as a significant internal mechanism to revitalize work efficiency. Against these backdrops, this study argues that prioritizing the safety needs of workers will improve work performance. These assumptions are reliably supported by the JD-R Model, hence, hypothesized that:

**Hypothesis** **1** **(H1).** *OHSM Practices positively influence work performance (i.e., safety performance, task performance).*

This hypothesis is expanded as:

**Hypothesis** **1a** **(H1a).** *OHSM Practices positively influence employees’ task performance.*

**Hypothesis** **1b** **(H1b).** *HSM Practices positively influences the safety performance of employees.*

#### 1.2.2. The Causal Link between OHSM Practices and Work Motivation

According to the Self-Determination Theory (SDT), individuals are naturally proactive in their eagerness towards personal growth and improvements hence exhibit psychological needs that are innate, universal, and significant for the maintenance of health [48]. People’s actions are determined by the interest they derive from it [49], hence, motivated employees mostly perform their job responsibilities if they intend to derive satisfaction or any form of benefits from what they do. As the oil and gas industry undisputedly remains an accident and injury-prone industry, it is expected that occupational health and safety needs, thus if satisfied or not satisfied by the company, are likely to induce or reduce work motivation. This can be as well-argued in line with the JD-R model, thus, the provision of effective health and safety systems as a job resource in such a risky work environment should meet the occupational health and safety job demands of employees. Past literature holds that the integration of the JD-R model has a direct impact on employees’ wellbeing and as well moderate and mediate the relationship between the characteristics of jobs and employees’ well-being. To wit, the absence of effective safety systems may cause job-related stress, burnout, psychological and health issues which will remain an impediment to job performance [50]. Based on these assumptions, the study anticipates that:

**Hypothesis** **2** **(H2).** *OHSM practices will determine work motivation among employees.*

#### 1.2.3. The Mediation and Moderation Influence of Work Motivation on the Causal Link between OHSM Practices and Work Performance

Over the years, the concept of Motivation was long considered as a major predictor of work performance [51]. This was clearly demonstrated in Campbell’s [33] model, which viewed motivation as one of the proximal factors that determines work performance. It is also assumed that individuals are likely to complete their tasks and exhibit positive work behaviours when they are intrinsically motivated [52]. According to Clarke [27], organizations must invest interest in providing workers with motivation factors that can provide an in-depth understanding of the linkage between organizational safety culture, safety behaviour, and safety outcomes. As presented by Vinodkumar and Bhasi [53], the motivation to practice safe behaviour in the workplace reduces accidents.

Hagger et al. [52], opine that individuals are likely to complete their tasks and work responsibilities when they are intrinsically motivated. Alternatively, extrinsically motivated employees mostly perform their job responsibilities in order to receive external rewards. Thus, people’s actions are determined by the interest they derive from it [54]. By this analogy, both intrinsic and extrinsic motivations are built based on the needs of the employees. Thus, employees become task-oriented if they find an interest and satisfaction in their work. In line with these empirical assumptions, the study further hypothesizes that:

**Hypothesis** **3** **(H3).** *Work motivation positively influences employees’ work performance.*

This hypothesis is further formulated as:

**Hypothesis** **3a** **(H3a).** *Work motivation positively influences employees’ task performance.*

**Hypothesis** **3b** **(H3b).** *Work motivation positively influences employees’ safety performance.*

Not least, the “protection motivation theory” describes motivation as the direct function of employees to work cautiously and protect them at the workplace. From the “Expectancy-value perspective theory”, thus, employees’ safety behaviours and intentions to adhere to safety management practices highly depend on the interest and the value the organization places on achieving good safety outcomes [55]. Both the “self-protection theory” and “Expectancy-value perspective theory” established the fundamental principle of employees ensuring self-safety during work, however, the “Expectancy-value perspective theory” extended the principle of motivation as an exchange of need between management and employees. Thus, in as much as employees are self-conscious about safety, they as well expect management to give critical attention to their safety needs to further drive their willingness towards work. In line with these assumptions, the

**Hypothesis** **4** **(H4).** *Work motivation will mediate and moderate the causal relationship between OHSM practices and employees’ work performance.*

Hypothesis 4 is further expanded as follows:

**Hypothesis** **4a** **(H4a).** *Work motivation will mediate the causal relationship between OHSM practices and employees’ safety performance.*

**Hypothesis** **4b** **(H4b).** *Work motivation will mediate the causal relationship between OHSM practices and employees’ task performance.*

**Hypothesis** **4c** **(H4c).** *Work motivation will moderate the causal relationship between OHSM practices and employees’ safety performance.*

**Hypothesis** **4d** **(H4d).** Work motivation will moderate the causal link between OHSM practices and employees’ task performance.

**Figure 1:**
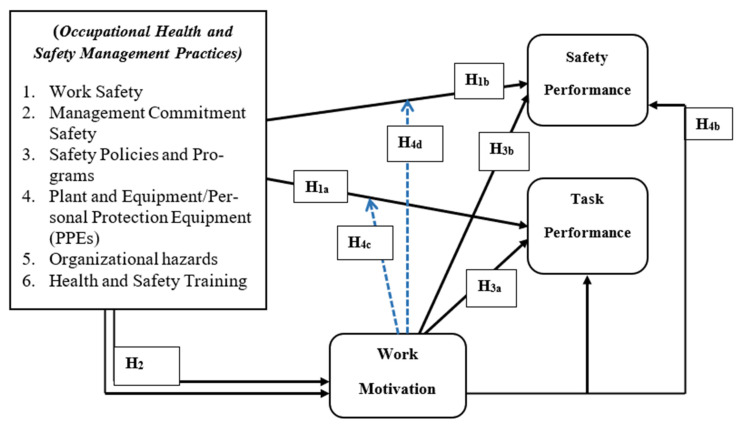
Conceptual Model.

Based on these hypotheses, the study built a conceptual model that combines the SDT and JD-R model to demonstrate how employees needs for and OHSM practices can trigger work performance through work motivation. As propounded by the JD-R model, both job resources and job demand is relative to the organizational structure and employees need just as the SDT focuses on the motivation behind the decisions people. Thus, one cannot undermine the fact that occupational health and safety needs remain one of the topmost needs among workers in highly risky industries, hence when satisfied, it may enhance interest, willingness and dedication to work. This assumption has been presented in the conceptual model in Figure 1 below:

## 2. Methods

### 2.1. Study Design

The present study design follows an exploratory research approach that utilizes robust quantitative research methods. A cross-sectional approach through the use of research questionnaires was utilized to solicit responses from participants. The exploratory study is appropriate for such a study because of the need for the study to investigate and clearly define the research hypothesis proposed.

### 2.2. Population and Sample

The study population was drawn from the Ghanaian oil and gas sector, which currently remains one of the largest economic contributors in Ghana. The sector is divided into several jurisdictions which include the upstream sector, mid-stream, and downstream sector. The population for the study, therefore, focuses on three (3) government-owned oil and gas companies which are directly involved in all five phases of oil and gas exploration and production. These include the Ghana National Petroleum Company (GNPC), Ghana National Gas Company (GNGC), and the Tema Oil Refinery (TOR). The GNPC falls under the upstream sector whiles GNGC and TOR fall under the downstream and midstream sector respectively. The activities of these organizations are divided into several units with different task objectives such as oil rig, explorations, mechanical maintenance, drilling, electrical installations, and machine operations among other serious tasks. The study however considered only workers whose work responsibilities are quite herculean and directly related to any of the phases of oil and gas production and exploration. These job types involved risky job tasks like drilling, pipe manoeuvring, pipe laying, and petrol chemical engineering

#### Sampling Technique and Data Collection Procedure

Three different sampling techniques, including purposive sampling, convenience sampling, and stratified sampling techniques were adopted for the study to select 1310 respondent groups using Krejcie and Morgan’s 1970 formula table for sampling.

With regards to the stratified sampling technique, the population was divided into several groups, and respondents were randomly and proportionally selected from each stratum. This strategy was very significant and appropriate for this study because, the respondents’ group for each of the three organizations proportionally varies, hence, there was the need to further divide them into subgroups with similar characteristics before casting the desired sample for the study. It is also important to state that, there exist different job categories in the three organizations with different levels of job risk. Respondents have therefore divided into four (4) strata as illustrated in Table 1. These four subgroups of participants were purposively selected following their level of knowledge in safety, working experience, and their direct involvement in risky work-related activities that demand safety attentions. The convenient sampling technique was finally utilized to cast a proportional sample size from each sub-category. The convenient sampling was preferred because the study recruited participants who fall within the strata and are readily available, accessible, and willing to participate in a study at the specified data collection period. Most responses were solicited through e-mails, the use of online survey tools and telephone calls. Some respondents were also recruited on the premises and worksites of the organization. In compliance with acceptable standards and research ethics, permission in a form of written letters was sought from the Human Resource departments, control and superseding officers of these organizations before proceeding to data collection. Respondents were oriented and assured that the data collected for the study will be confidential and used for academic purposes only. All respondents’ were willingly allowed to respond to questions at their own convenient and appropriate time. No respondent in the entire study was oppressed, punished, or given any form of financial reward to accept or deny partaking in the study. The traditions and values of the sampled population were strictly followed. The entire data lasted for a period of 18months, with a response rate of 93%.

Table 1 below presents brief information on the study’s population and sampling techniques:

### 2.3. Measures

All the scales adopted for this study went through pilot testing and minor modifications were made to fit the context of the study. All ambiguous and double-barreled questions were avoided for precision and clarity. Only scales that met the reliability test criterion through an exploratory factor analysis were used for further data analysis.

#### 2.3.1. OHSM Practices

Considering the ongoing debate on a generally established and effective instrument to measure OHSM practices, this study, therefore, combines different industry-specific occupational health and safety scales published only in the top quartile journals in occupational health and safety management with reliabilities greater than 0.80. The OHSM practices contextualize six distinct dimensions scale that assesses the general employees’ perceptions on organizational safety issues. These include Work Safety, Management’s commitment to safety, Safety programs/policies [56]; Organizational Hazards, Plant and Equipment’s/Personal Protection Equipment, Health and Safety Training [57,58].

#### 2.3.2. Work Performance

The work performance variable unlike previous performance constructs was assessed using safety performance and task performance. This study anticipates that the promotion of positive safety behaviour while performing various responsibilities at work is likely to enhance effective work engagements, hence, improves task-related activities. The task performance scale was adopted from the study of Abramis (1994) and the safety performance scale from the study of Griffin and Neal [13]. Empirical studies have shown that workers who exhibit positive safety behaviour are more productive than those who do not [13,59,60].

#### 2.3.3. Work Motivation

The Multidimensional Work Motivation Scale (MWMS) which was developed and built on the concept of SDT was adopted as the work motivation variable. The SDT has been used to study the relationship between motivational mechanisms and job engagements among employees [61,62]. Unlike most previous motivation measurement scales built on the SDT, thus; assessing why employees do an activity through the rating of statements describing the several types of behaviours, [63,64,65]; this current MWMS focuses more on assessing why employees “would” or “do” put a certain degree of efforts into their work.

#### 2.3.4. Items Measurement

The measurement scale for all variables was assessed on a 5-point Likert scale ranging from 1–5, where 1 represents strongly Disagree and 5 represents Strongly agree to the items. This is specified in Table 2 below:

### 2.4. Method for Data Analysis

Structural equation modelling (SEM) was used as the main method of data analysis. Specifically, the second-order factor model method was preferred. This method produces robust inferences relative to the traditional regression model. Descriptive and reliability analysis was initially performed to ascertain the means, standard deviation and Cronbach’s alpha of all scales. The Kaiser–Meyer-Olkin Measure of Sampling Adequacy (KMO-MSA) and Bartlett’s Test of Sphericity (BTS) was as well estimated. Consistent with prior works of Mardani et al. [66], the study set a strict level of significance for the regression coefficients at (95%) for each latent variable and accordingly evaluated the reliability, validity, and internal consistency of each latent variable. Fit indices were further used as an indication to ascertain whether the model is acceptable or otherwise. Thus, the Chi-square, Root Mean Square Error of Approximation (RMSEA), comparative fit index (CFI), Standardised Root Mean Residual (SRMR), Tucker Lewis index (TLI), and Goodness of Fit (GFI) were closely monitored.

The procedures of Baron and Kenny’s [67] bootstrapping method, which assesses the indirect effect of the mediating variable and the methods of Hayes [68] PROCESS macro procedures were followed to estimate the mediating and moderating effect respectively. Sobel [69] suggested that, to identify a full or partial mediation the reduction in variance explained by the independent variable must be significant as determined by one or numerous tests. The study, therefore, adopted the estimation of the variation accounted for (VAF) as well. The Statistical Product and Service Solutions (SPSS) version 26.0 (IBM, Armonk, NY, USA) was used for data coding, descriptive, reliability and validity analysis whiles the path estimates and confirmatory factor analysis were all estimated through the use of version 25.0 of Analysis of a Moment Structures (AMOS).

## 3. Results

### 3.1. Description of Respondents

The majority of the respondents recruited for the study can be defined as skilful male participants between the age of 26–45 years with working experience ranging from 3–5 years. The skilful level of respondents in this study measures respondents’ degree of knowledge about their job, the risks or dangers involved, the technical ability, ability to understand and combat work challenges, and the exceptional use of work-related tools. Table 3 below gives further details on the respondents recruited for the study.

### 3.2. Descriptive and Reliability Analysis

The results as presented in Table 4 below indicate that the Kaiser–Meyer-Olkin Measure of Sampling Adequacy (KMO-MSA) of the scales were all above the 0.600 thresholds. The scale measuring work performance indicated the highest KMO-MSA of 0.815 whiles the work motivation scale indicated the lowest of 0.775. Additionally, Bartlett’s Test of Sphericity (BTS) for the scales were all significant. The reliability statistics as well indicated Cronbach’s α greater than the threshold of 0.70 for all constructs.

### 3.3. Convergence Validity

The results for the AVE and CR for all constructs as presented in Table 5 satisfied all conditions of a convergent validity test. Thus, the AVE and CR were above the threshold of 0.5 and 0.7 respectively.

### 3.4. Discriminant Validity

The findings as indicated in Table 6 below show that the scales are distinct and unrelated as the square root of AVE for each scale was higher than their correlation with other scales. This simply confirms that the items are unique and do not discriminate against their measurement construct [70].

### 3.5. Goodness of Fit Index and Structural Equation Model

**Figure 2:**
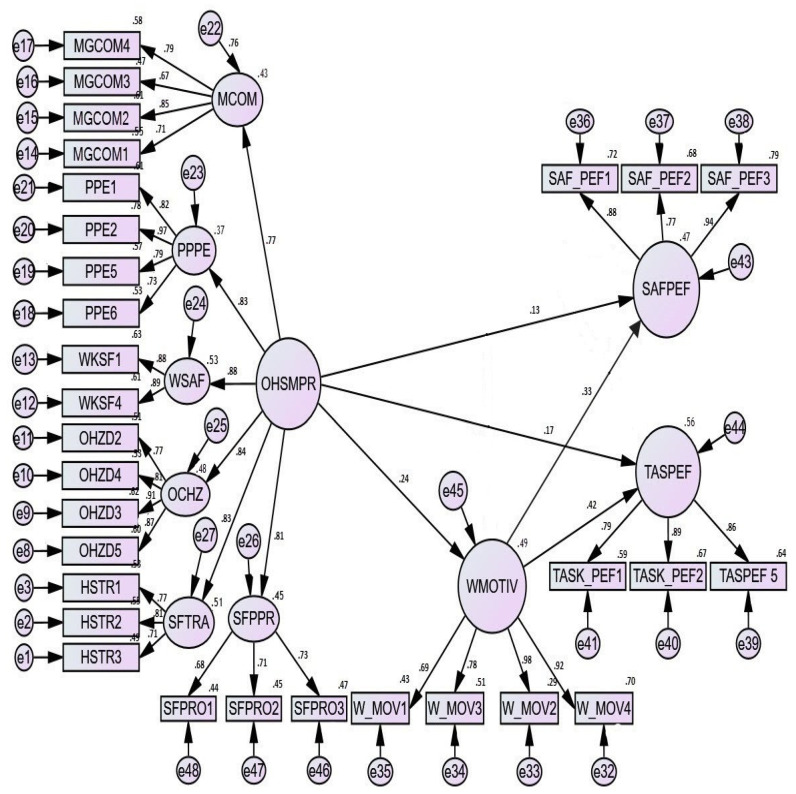
Structural Equation Model.

The results for the Structural Equation Model and Goodness of Fit Index is indicated in Figure 2 and Table 7 below:

The results for all the model fit indices as indicated in Table 7 above satisfy the statistical criteria for the structural equation model.

### 3.6. Path Analysis

**Figure 3:**
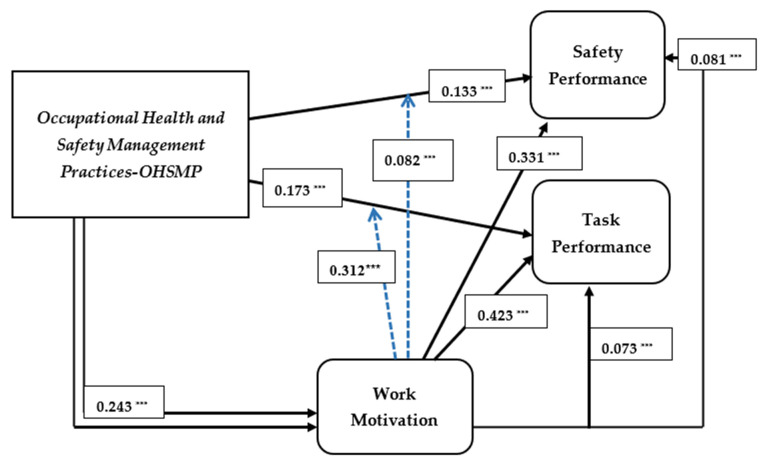
Path Analysis. Note: *** significant at 95% confidence interval.

The results of the path analysis as presented by SEM were used to determine if the formulated hypothesis as proposed in accordance with the conceptual model should be accepted or rejected using the *p*-values of the path estimates which were tested at a 95% confidence level. Figure 3 below illustrates the results for all path estimates, including the mediating and moderating effects.

The SEM results show that all paths explaining the causal relationship between the latent constructs were positively significant at 95% confidence levels. The path explaining the relationship between the main exogenous variable, OHSM practices, and the endogenous variable work performance indicated a significant relationship of 0.137 between OHSM practices and safety performance and 0.173 between OHSM practices and task performance. These results also show that OHSM practices have a higher influence on employees’ task performance than safety performance. Thus, the basis of these findings anticipates that the degree of workers’ ability to conform to safety procedures and undertake safety initiative whiles performing their various tasks and as well the ability to perform effectively is determined by the degree of OHSM effectiveness at the workplace.

The path estimates as well indicated that OHSM practices significantly influence employees’ work motivation, as 0.247 was estimated between OHSM practices and work motivation. It is important to highlight that, the path estimates between OHSM practices and work motivation are higher than path estimates the influence OHSM practices have on both task and safety performance.

Concerning the path estimates showing the causal links between work motivation and work performance, the results indicated that work motivation influences both safety performance and task performance at 0.331 and 0.423 respectively. Thus, the findings denote that the degree of task accomplishment at work, safety compliance, and safety participation among employees is likely to be influenced by employees’ work of motivation. Clearly, the estimated paths depict that work motivation affects worker task performance more than safety performance. Be as it may be, work motivation is a significant and positive predictor of work performance as hypothesized in the model. The results of the path estimate are presented in Table 8 below:

### 3.7. Mediation and Moderation Analysis

Fundamentally, the significance of the mediation model is to identify and describe the channel or process that underlies the observed association between an exogenous and endogenous variable. The results of the mediation effect of work motivation are therefore presented in Table 9 below.

As estimated by the bootstrapping method, the indirect effect estimates predict that work motivation significantly and partially mediates the influence OHSM practices have on both safety performance and task performance. Thus, the indirect effect indicated 0.083 as the effect of work motivation on the causal link between OHSM practices and safety performance, while it indicates 0.092 for OHSM practices and task performance. The results as estimated by the indirect effect also show that work motivation has a greater effect on the relationship between OHSM practices and task performance as compared to OHSM practices and safety performance. The VAF similarly indicated that work motivation explains 51.5% of the variations in the effect of OHSM practices on safety performance, and as well as 58.7% of the variations in the causal link between OHSM practices and task performance.

On the other hand, the moderating effect seeks to understand the interaction variable that affects the direction or strength of the relationship between dependent and independent variables. Moderators can strengthen, weaken, or reverse the nature of a relationship hence the need to understand the moderating influence of work motivation. The results are presented in Table 10 below.

The moderation results as presented in Table 10 show that work motivation improves the strength of the influence OHSM practices have on both task performance and safety performance which are both indicators for work performance. Thus, the results show that work motivation has a significant moderating effect of 0.0817 and 0.3128 on the influence of OHSM practices on safety performance and task performance respectively. The causal strength of OHSM practices on both safety performance and task performance significantly increased compared to what was estimated by the SEM path estimates after accounting for these moderations. These results, therefore, show that work motivation may improve the strength between OHSM practices and work performance in the Ghanaian oil and gas sector. Base on the estimated results from the moderation analysis, the hypothesis 4c and 4d is as well accepted.

## 4. Discussions

As declared, the current study seeks to understand the moderating and mediating influence of work motivation in the causal link between Occupational Health and Safety Management (OHSM) practices and work performance through a cross-sectional study in the Ghanaian oil and gas sector. The findings of this study established a positive and significant causal link between OHSM practices and task performance and safety performance, however, OHSM practices have a higher influence on task performance than on safety performance. As well, OHSM practices were found to significantly influence work motivation higher than both safety and task performance. The path estimates further found work motivation as a positive predictor of both task and safety performance whiles it also significantly moderates and partially mediates the causal link OHSM practices have with both task and safety performance. These findings basically show that the degree of workers’ ability to conform to safety procedures and undertake safety initiative whiles performing their various tasks and as well the ability to perform effectively is determined by the degree of work motivation and OHSM practice effectiveness at the workplace. Katsuro et al. [71] found the absence of good safety management practices to negatively influence the production efficiency of workers. Previous studies as well report that the lack of safety compliance is a significant predictor of workplace accidents and the major cause of work disasters, specifically in the oil and gas sector [27,72,73]. This also corroborates with the yearly assessment of incident data reported by the International Association of Oil and Gas Producers (OGP), which reported that safety violations due to the refusal to comply with safety procedure remain a common factor causing serious catastrophes and accidents among oil and gas organizations [74,75,76].

Models and theories propounded by [77,78,79,80] have all built empirical evidence that supports the linkage between OHSM, employee wellbeing, productivity, and organizational performance. The finding further confirms the study of Fernández-Muñiz et al. [28] who identified Health and Safety Management Systems as a significant approach to enhance business transparency, competitiveness, and performance whiles it as well agrees with the results of Gopang et al. [42] who predicted a significant relationship between health and safety systems and performance. Ramazan et al. [44] also revealed that the application of safety procedures, strategic risk mitigation techniques, application of safety rules, and management safety support significantly influences productivity levels among employees. As organizations improve their working environment, accidents and injuries are reduced and performance is improved [53].

A motivated worker is a happy worker and a happy worker performs better. Thus, organizations should strive to pursue the interest of workers by understanding and providing needs that can initiate and induce their work interest at all times. Literature has established that motivation factors are significant and important initiators of employee work engagement. As established in the findings of Hagger et al. [52] individuals are likely to complete their tasks and work responsibilities when they are intrinsically motivated. Alternatively, extrinsically motivated employees mostly perform their job responsibilities in order to receive external rewards. Thus, people’s actions are determined by the interest they derive from it [49]. Focusing on the mediating and moderating influence of work motivation on the causal link between OHSM and work performance, the indirect mediating effect and moderating effect basically means that the significant effect of OHSM practices on task and safety performance as predicted by the SEM path analysis is partly explained by the degree of work motivation among workers. With a specific focus on the moderating effect, the finding indicates that the higher the motivational levels of workers, the higher the influence of OHSM practice on work performance and vice versa. These findings agree with previous studies that demonstrated that effective safety systems and policies improve employees’ level of connection with their working environment. This idea can also be attributed to the self-determination theory [81,82]. The findings can as well be associated with the assumptions of the conceptual framework and research theories proposed in the study. Thus, organizations need to pursue and view OHSM practices as a significant work resource that can induce work engagements among employees.

### 4.1. Theoretical Implications

In this current study, the concept of motivation and its significance in safety management was well-grounded in the study’s hypothesized model. Thus, the study shifted and expanded the argument of Neal and Griffin [14,53] by presenting a holistic view on the concept of motivation and its influence on work performance. In most risky organizations, such as the oil and gas sector, organizational failures to efficiently promote workplace safety are very common [22]. Hence, integrating theories such as the perceived organizational support for safety, JD-R model, and Self Determination Theory, to develop a single hypothesized model in this study gives a holistic view on the significance of human perceptions and attitudes towards work as an integral factor that can be shaped to ascertain the best performance outcome through systematic OHSM practices. Thus, juxtaposing the research findings to the assumptions advanced in the hypothesized conceptual model, effective OHSM practices in the Ghanaian oil and gas sector may be assumed to be a job resource that is likely to stimulate employees’ interest, dedication, work engagements, satisfaction in performing work responsibilities and promote good safety behaviour.

The JD-R model posits that work engagements are improved when essential resources are provided for employees as a safe working environment predicts positive working behaviour that is likely to enhance organizational efficiency [11,71,83]. To be more precise, the study through the findings opines that understanding human behaviour can easily pave the way for management to ascertain the motivating factors of employees and pursue them accordingly, specifically, those related to the improvement of occupational health and safety management practices. Thus, although enhancing safety performance is an obvious reason to improve OHSM practices, other variables, in the case of this study, work motivations come into play in the emergence of a new approach towards OHSM practices. Systematic OHSM practice is undoubtedly a turning point in improving safety and quality of life at the workplace, and its emergence remains a pivotal mechanism to achieve an industrial revolution, hence, its context must look beyond what the past experience teaches us about safety.

To crown it all, the study employs a wider perspective of OHSM practices from both organizationally and employee-centred approaches as important components of understanding the safety-performance nexus. According to Veltri et al. [84], when occupational safety is examined in the wider perspective as the rationale for improving performance, safety becomes paramount. The study, therefore, adds to the existing literature by expanding on theories that can enhance positive work outcomes. To the best of our knowledge, unlike safety motivation, this study is the first attempt to integrate work motivation as a universal concept of motivation theory as hypothesized in the model. Moreso, the mediating and moderating effects of work motivation as established in the findings were found to be a significant contribution and expansion of literature.

### 4.2. Practical Implications

The oil industry contains many hazardous risks that may cause both incidents and major disasters, yet, most organizations in this sector underestimate the effect of poor OHSM practices on performance due to the lack of understanding and application of effective administration of OHSM [85,86,87]. In Ghana, most industries face issues such as the lack of safety policy directions, poor safety infrastructure, lack of funding for safety systems, inadequate OHS work-related accidents or injuries monitoring mechanism, inadequate basic health and safety equipment and protective wears, non-existence of continuous safety training policies, employee’s apathy towards abiding by safety rules, and the lack of effective implementation of risk elimination plans [88,89,90]. Organizations that trade OHSM for short-term operational benefits put not only their employees at risk but also their business and performance [84].

The findings of the study provide new knowledge that challenges organizations to develop industry-specific safety measures that will escalate safety and task performance among workers in highly risky industries. This can be done through frequent employee engagements, involving employees in safety plans, strict monitoring of safety programs, and the ability to exhibit high levels of safety commitment and leadership by employers and supervisors. As one of the basic assumptions of the social exchange theory by Homan [91], human behaviour is influenced by the exchange of activities, hence, it is expected that employees who benefit from a safe working environment are likely to be highly committed to task accomplishment [44]. In line with these assumptions, predicting factors of safety performance should be an utmost concern to organizations. Similarly, as work performance is significantly associated with OHSM practices, it sheds light on the significant effect safety values and norms have on workers’ efficiency. This means that to achieve the best outcomes, safety and task performance among workers should be tied with OHSM practices.

The significant influence of work motivation on both safety and task performance which are indicator measurements for work performance established in these findings also supports the theories propounded in the JD-R model. Which is, the balance between job demand and job resources promotes the health and wellbeing of employees at the workplace. This literally means that the prioritization and investment in systematic occupational safety and health systems may provide a balanced, comprehensive approach to hazard control and as well promote employees’ wellbeing. It is as well expected that the appropriate supporting work environment can promote work engagements, trigger work motivation, and enhance work performance [92,93,94]. This is also similar to the SDT, which describes the relationship between motivational mechanisms and job engagements among employees [61,62]. Based on this empirical evidence, the study opines that providing incentives for safety compliance and safety participation can drive work motivation and enhance productivity. As posited by Ryan and Deci, [48] people are more appreciative, receptive, and obedient when their interests are met.

As posited by the POSS theory, workers’ safety attitudes are influenced by the level of management’s interest in employees’ health and safety needs. This current study, therefore, expresses the view that measures such as discussing and ascertaining the practicality and basis for management and frontline supervisors’ to be committed towards the motivating of employees to engage in safe work behaviour. Instead of annual reviews, safety officers must periodically review the extent to which the annual safety plans have improved safety and work performance. Thus, management and safety leaders must constantly and frequently monitor safety plans and the compliance to safety systems by employees. Putting workers in safety teams, recognizing and rewarding safety behaviours, promoting workers that exhibit positive safety attitudes, and engaging workers in safety decisions at the top level should be given much priority, as the degree of employee performance is often determined based on their enthusiasm, motivation, and competence [95]. These are very significant remedies that are likely to transcend work performance through work motivation.

Finally, improving workers’ health and safety is not only an organizational issue but as well the government. The health of a nation is dependent on the health of the people. hence, the government has a crucial role to play in the protection of life and properties. At the legislation level, an appropriate system of governance must be established to support, monitor, and enforce the successful implementation of safety programs as an integral part of management systems. It is expected that a continuous safety commitment and enforcement at the national front together with safety awareness and management commitment towards safety at the industry level can improve the degree of OHSM practices in the oil and gas sector, including other risky industries.

### 4.3. Limitations of the Study

The strength of this study is built on the robustness of research methodologies adopted, as they were all built on hypotheses that are well-supported by theories. On this basis, the study’s methodology design has a strong foundation for application in a new context. The study as well focused on variables with strong predictive capacities of work performance and investigated the OHSM—work performance nexus among the cross-sectional workforce using a rigorous quantitative approach by presenting a comprehensive understanding of the research issue. Irrespective of the reliability and validity of this study, some items measuring the latent constructs were slightly modified and grammatically restructured to improve clarity and easy understanding. The educational level, age, and experience among respondents are different hence the interpretation of question items and responses given is likely to be affected by these demographic factors.

Again, the cross-sectional nature of the study is not enough to justify the satisfaction of the results revealed by the study. Thus, the study sampled only government-owned oil and gas organizations in Ghana, hence, the views and reflections of privately owned oil and gas companies were not sorted, this may not reflect the general view of the entire oil and gas sector in Ghana as private organizations may have a different approach towards OHSM administration. This does not however reduce the reliability and quality of the findings in this study as the government-owned oil and gas organizations control the production and regulatory activities of the sector.

The study is as well subjected to limitations such as socially desirable and modal responses due to the self-report measures adopted for data collection. Other mediating factors such as work pressure and emotional exhaustion can be considered to strengthen the claims on the current results. Despite these limitations, the study results are expected to be relevant to the development and improvement of occupational health and safety policies, procedures, and standards that can improve work performance through workers’ motivation.

Finally, there is a need to reiterate that this current study expands the safety literature, not only by providing a rare case study on the importance of health and safety interventions but also showing quantitative evidence for strengthening the safety-performance nexus through work motivation. Based on the context of this study, future research should therefore focus on expanding the research designs, by including qualitative methods to expand the theories propounded in the study. This will improve the research results and provide a deeper perspective of the concepts and ideas presented in this study. Factors such as safety commitment and safety leadership can reliably be measured effectively through management responses, however, this study side-lined safety supervisors and employees in managerial positions. To holistically address the research issue, future studies can therefore focus on a cross-sectional study that integrates the views of both supervisors and managers. It was also observed that variables such as emotional intelligence, psychological stress and anxiety, employee resilience and work altruism have not yet received maximum attention in the safety-performance relationship, hence, can be explored in future studies.

### 4.4. Conclusions

Employees’ health and safety at the workplace are both psychological and emotional concern to the worker as it is directly linked to the quality of life. Individuals are naturally proactive and intuitively rational in their eagerness towards personal growth and improvements, hence, may exhibit psychological needs that are innate, universal, and significant for the maintenance of their health. As demonstrated by this current study, work motivation is a significant conduit that improves the safety-performance nexus in the oil and gas sector. Undoubtedly, the existence of systematic and efficient health and safety management systems and practices do not only empower organizations to control the several risk exposures they encounter but as well influence employees’ behaviour and attitude towards work. Considering the importance of OHSM practices and their related positive outcomes it alludes to work performance, the study needs to highlight that organizations must link safety leadership and safety commitment in all aspects of safety administration. These are very significant remedies that are likely to yield the expected safety outcomes, performance and eagerness towards positive work attitudes.

## Figures and Tables

**Table 1:** Population and Sample Distribution of respondents.

Phase of Production	Activities	Respondents	Krejcie and Morgan’s (1970)	Data Collected
Upstream sector	Exploration Drilling Production Engineering and construction			
		Labourers	116	116
		Technicians	118	112
		Machine Operators	118	112
		Engineers	116	114
		
		Total	468	454
Downstream sector	Preparation Refining Processing Purifying			
		Labourers	116	98
		Technicians	118	112
		Machine Operators	118	110
		Engineers	116	104
		
		Total	468	424
Midstream sector	Storage Pipelining Oil and gas transfer Transporting Natural gas gathering and processing Distribution			
		Labourers	116	110
		Technicians	118	108
		Machine Operators	118	110
		Engineers	116	104
		
		Total	468	432

**Table 2:** Scale Items Dimensions.

Study Variables	References	Explanation
**Endogenous**		
*Work Performance (WP)*	Abramis (1994)	i. Safety Performance (4 items)
	Griffin and Neal (2000)	ii. Task Performance (5 items)
**Mediating Variables/** **Exogenous**		
*Work motivation (WM)*	Gagné et al. (2015)	The Multidimensional Work Motivation Scale (MWMS) (5 items)
**Exogenous**		
*Occupational Health and Safety Management (OHSM) Practices*	Hayes et al. (1999); Cox and Cheyne, (2000); Choudhry, Fang, and Ahmed (2008)	i. Work Safety (4 items)
		ii. Management’s commitment to safety (5 items)
		iii. Safety Programmes/Policies (5 items)
		iv. Organizational Hazards (4 items)
		v. Plant and Equipment/Personal Protection Equipment (6 items)
		vi. Health and Safety Training (4 items).

**Table 3:** Description of Respondents.

Gender	Frequency	Percentage
Male	1042	79.5
Female	268	20.5
Total	1310	100
Age		
Under 25 yrs	130	9.9
26–35 yrs	604	46.1
36–45 yrs	456	34.8
46–60 yrs	120	9.2
Total	1310	100
Education	Frequency	Percent
Junior High School	324	24.7
Senior High School/Technical education	428	32.7
Bachelors Degree and Professional Certification	558	42.6
Total	1310	100.0
Work Experience		
Under 1 year	32	2.4
1–2 years	106	8.1
3–5 years	916	69.9
6–10 years	136	10.4
over 10 years	120	9.2
Total	1310	100.0
Job Skills		
Not Skillful	-	-
Somehow skilful	-	-
Skilful	1166	89.0
Very Skillful	136	10.4
Extremely Skillful	8	0.6
Total	1310	100.0

**Table 4:** Results for Reliability Analysis.

Exogenous Variables	Items	Mean	Std. Deviation	Cronbach’s α	KMO	BTS Sig.
**OHSMPR**	29	3.020	0.533	0.708	0.811	0.000
1. Work Safety (WSAF)	4	3.468	0.249	0.706		
2. Management Commitment to Safety (MCOM)	5	3.787	0.605	0.765		
3. Safety Programs/Policies (SAFPO/PR)	5	2.984	0.539	0.831		
4. Health and Safety Training (HSAFT)	5	2.863	0.563	0.837		
5. Plant and Equipment/Personal Protection Equipment (PPEs) (PPPE)	6	2.360	0.622	0.786		
6. Organizational hazards (OGHZ)	4	2.660	0.602	0.879		
**Endogenous Variables**						
**Work Performance**	9	3.430	0.489	0.772	0.815	0.000
Safety Performance (SAFPEF)	4	3.112	0.263			
Task Performance (TASPEF)	5	3.748	0.714			
**Mediating—Moderating Variables**						
Work motivation (WMOTIV)	5	3.249	0.547	0.855	0.775	0.000

**Table 5:** Results of Convergence Validity of Scales.

OHSM Practices—Exogenous	AVE	CR
1. Work Safety Scale	0.695	0.957
2. Management’s Commitment to Safety Scale	0.707	0.896
3. Safety Programmes/Policies Scale	0.688	0.930
4. Plant and Equipment/Personal Protection Equipment (PPEs) Scale	0.698	0.965
5. Organizational Hazards Scale	0.712	0.813
6. Health and Safety Training Scale	0.762	0.869
**Endogenous—Work Performance**		
1. Safety Performance Scale	0.687	0.824
2. Task Performance	0.642	0.781
**Mediating and Moderating**		
Work Motivation	0.727	0.827

**Table 6:** Results of Discriminant Validity.

OHSMPR	WSAF	MCOM	SFPRO	PPE	OHZD	HSTR
WSAF	**(0.834)**					
MCOM	0.521 ***	**(0.841)**				
SFPPR	0.477 ***	0.591 ***	**(0.829)**			
PPPE	0.545 ***	0.453 ***	0.388 ***	**(0.835)**		
OCHZ	0.442 ***	0.521 ***	0.423 ***	0.423 ***	**(0.843)**	
SFTRA	0.539 ***	0.456 ***	0.511 ***	0.405 ***	0.485 ***	**(0.872)**
WPERF	SAFPEF	TASPEF				
SAFPEF	**(0.829)**					
TASPEF	0.521 ***	**(0.801)**				

*** Significant at 95%, note: figures in bold represent the square root of the AVE.

**Table 7:** Results of Goodness of Fit for SEM.

Fit Indices	Results	Criteria
$\chi^{2}/\mathrm{df}$	4.393	<5
GFI	0.951	>0.80
SRMR	0.031	<0.08
RMSEA	0.048	<0.08
NFI	0.953	>0.80
CFI	0.979	>0.80
TLI	0.957	>0.80

**Table 8:** Path Estimates of SEM.

Hypothesis	Paths	Standardized Estimates	S.E.	C.R.	*p*-Value	Decision
H1a	TASPEF<---OHSMPR	0.173 ***	0.069	2.507	0.000	Accepted
H1b	SAFPEF<---OHSMPR	0.133 ***	0.024	5.541	0.000	Accepted
H2	WMOTIV<---OHSMPR	0.243 ***	0.098	2.479	0.007	Accepted
H3a	TASPEF<---WMOTIV	0.423 ***	0.033	12.818	0.000	Accepted
H3b	SAFPEF<---WMOTIV	0.331 ***	0.042	7.880	0.000	Accepted

*** 95% significance level.

**Table 9:** Mediation Analysis.

Mediator	Path Estimates	Estimate	*p*-Value	Decision
**WMOTIV**	WMOTIV<---OHSMPR	0.243	0.000	
	SAFPEF<---WMOTIV	0.331	0.000	
	TASPEF<---WMOTIV	0.423	0.000	
Direct Effect	SAFPEF<---OHSMPR	0.133	0.000	
**Indirect Effects**	SAFPEF<---WMOTIV<---OHSMPR	**0.081**	**0.037**	H4b: Accepted
Total Effects		0.213	0.000	
	**Variation Accounts For (VAF)**	**0.515**		Accepted
Direct Effect	TASPEF<---OHSMPR	0.173	0.000	
**Indirect Effects**	TASPEF<---WMOTIV<---OHSMPR	**0.073**	**0.037**	H4a: Accepted
Total Effect		0.246	0.005	
	**Variation Accounts For (VAF)**	**0.587**		Accepted

**Table 10:** Moderating Analysis.

Dependent Variable: SAFPEF						
	**Co-efficient**	**Se**	**t**	***p*-Value**	**LLCI**	**ULCI**
Constant	3.9712	0.7621	5.2109	0.0019	1.4747	2.4676
OHSMPR	0.5063	0.2439	2.0758	0.0083	0.9853	1.0274
WMOTIV	0.1489	0.1481	1.0052	0.0052	0.4398	0.9142
**Int_1**	**0.0817**	0.0488	1.6729	0.0048	0.0142	0.1776
**Dependent Variable: TASPEF**						
	**Co-efficient**	**Se**	**t**	***p*-Value**	**LLCI**	**ULCI**
Constant	6.6736	1.1780	5.6654	0.0000	4.3606	8.9867
OHSMPR	0.4963	0.3770	3.9687	0.0001	0.2366	0.7560
WMOTIV	0.1863	0.2290	3.1720	0.0016	0.1759	0.2767
**Int_1**	**0.3128**	0.0755	4.1443	0.0000	0.1646	0.4611

Se—Standard error, t—t statistic, LLCI—95% confidence interval Lower boundary, ULCI—95% confidence interval Upper boundary.

## Data Availability

Data is available upon request from researchers who meet the eligibility criteria. Kindly contact the first author privately through the e-mail.
